# A Study of Reasons for Self-Disclosure on Social Media among Chinese COVID-19 Patients: Based on the Theory of Planned Behavior Model

**DOI:** 10.3390/healthcare11101509

**Published:** 2023-05-22

**Authors:** Yi Wang, Tianrui Qiao, Chao Liu

**Affiliations:** 1 College of Journalism and Communication, Huaqiao University, Xiamen 361021, China35355@hqu.edu.cn (T.Q.); 2 Business Analytics Research Center, Chang Gung University, Taoyuan 33302, Taiwan

**Keywords:** COVID-19, self-disclosure, Theory of Planned Behavior

## Abstract

Background: With a massive population of internet users, China has witnessed a shift in the behavior of social media users towards the COVID-19 pandemic, transitioning from reticence to frequent sharing of information in response to changing circumstances and policy adjustments of the disease. This study aims to explore how perceived benefits, perceived risks, subjective norms, and self-efficacy influence the intentions of Chinese COVID-19 patients to disclose their medical history on social media, and thus to examine their actual disclosure behaviors. Methods: Based on the Theory of Planned Behavior (TPB) and Privacy Calculus Theory (PCT), a structural equation model was constructed to analyze the influence paths among perceived benefits, perceived risks, subjective norms, self-efficacy, and behavioral intentions to disclose medical history on social media among Chinese COVID-19 patients. A total of 593 valid surveys were collected via a randomized internet-based survey, which constituted a representative sample. Firstly, we used SPSS 26.0 to conduct reliability and validity analyses of the questionnaire, as well as the tests of demographic differences and correlations between variables. Next, Amos 26.0 was employed to construct and test the model fit degree, identify the relationships among latent variables, and conduct path tests. Results: Our findings revealed the following: (1) There were significant gender differences in the self-disclosure behaviors of medical history on social media among Chinese COVID-19 patients. (2) Perceived benefits had a positive effect on self-disclosure behavioral intentions (β = 0.412, *p* < 0.001); perceived risks had a positive effect on self-disclosure behavioral intentions (β = 0.097, *p* < 0.05); subjective norms had a positive effect on self-disclosure behavioral intentions (β = 0.218, *p* < 0.001); self-efficacy had a positive effect on self-disclosure behavioral intentions (β = 0.136, *p* < 0.001). (3) Self-disclosure behavioral intentions had a positive effect on disclosure behaviors (β = 0.356, *p* < 0.001). Conclusions: Our study, by integrating TPB and PCT to examine the influencing factors of the self-disclosure behaviors among Chinese COVID-19 patients on social media, found that perceived risks, perceived benefits, subjective norms, and self-efficacy had a positive influence on the self-disclosure intentions of Chinese COVID-19 patients. We also found that self-disclosure intentions, in turn, positively influenced disclosure behaviors. However, we did not observe a direct influence of self-efficacy on disclosure behaviors. Our study provides a sample of the application of TPB in the context of social media self-disclosure behavior among patients. It also introduces a novel perspective and potential approach for individuals to address the feelings of fear and shame related to illness, particularly within the context of collectivist cultural values.

## 1. Introduction

In March 2020, the World Health Organization (WHO) classified the outbreak of novel coronavirus pneumonia (COVID-19) a “global pandemic”. According to the *Law of the People’s Republic of China on Prevention and Treatment of Infectious Diseases*, COVID-19 was identified as an acute respiratory tract infection and controlled as a Class A infectious disease in China, which requires isolation in facilities with effective isolation and protective measures.

On 7 December 2022, China’s State Council Joint Prevention and Control Mechanism Integrated Group revised the epidemic control policies, permitting asymptomatic infected individuals and mild cases to undergo home isolation if they meet certain conditions or choose centralized isolation voluntarily. On 26 December 2022, the National Health Commission of China renamed the disease as “novel coronavirus infection”. From 8 January 2023, a “Category B” policy will be implemented for disease control [1], which entails that infected patients will no longer be subjected to isolation measures and close contacts will no longer be traced. Following the revision of China’s COVID-19 prevention and control policy, the Chinese Center for Disease Control and Prevention reported on 22 December that the total number of confirmed cases in China had reached a peak of 6.94 million.

The 51st Statistical Report on the Development of the Internet in China by China Internet Network Information Center (CNNIC) revealed that as of December 2022, the number of internet users in China has reached 1.067 billion, indicating a 75.6% internet penetration rate. Additionally, the per capita weekly Internet usage time has been up to 26.7 h [2].

As social media communication continues to grow in influence [3], an increasing number of Internet users are inclined to express themselves on WeChat, QQ, and other social media platforms. 

During the COVID-19 epidemic, there has been a rise in the number of users who have shared more personal experiences with the disease on social media platforms such as WeChat and Weibo [4], especially following the optimization and adjustment of the isolation policy. A large number of users have disclosed their medical history with COVID-19 on social media, including their time of diagnosis and their feelings of the infection.

Drawing on the aforementioned context, this study aims to investigate the reasons that underpin the decisions of COVID-19 patients to disclose their illness on social media. Grounded in the Theory of Planned Behavior and the Privacy Calculus Theory, this research endeavors to construct a structural equation model to examine the factors prompting Chinese COVID-19 patients to transition from concealing to openly revealing their medical status and history on social media.

## 2. Literature Review and Research Hypothesis

### 2.1. Self-Disclosure

Self-disclosure, referring to the voluntary act of revealing various aspects of oneself, including but not limited to descriptive information, emotions [5], and thoughts [6], is considered as a crucial coping behavior in both institutionalized therapeutic [7]. Individuals carefully weigh the pros and cons of disclosure and assess potential benefits and risks before deciding to reveal personal information [8]. For instance, users of online health communities may share their personal information to seek guidance on disease prevention or treatment [9,10], or to share medical histories, experiences, and feelings [11] with others who are undergoing similar experiences to receive emotional support.

Self-disclosure is a complex behavior that can be affected by external social environments or internal psychological or personal traits [8]. Its motivation is multifaceted. However, research on factors influencing the self-disclosure behavior of social media users is scattered and lacks uniformity [12], with China lagging behind other countries in this regard [13].

Most of the existing studies on self-disclosure behavior of social media users have employed theories such as Privacy Calculus Theory (PCT) [14,15], Theory of Planned Behavior (TPB), Theory of Reasoned Action (TRA) [16], Social Cognitive Theory [17], Uses and Gratifications Theory (UGT) [18], Social Exchange Theory [8,19], Attachment Theory [20], and Theory of Personality Traits [21].

The Theory of Planned Behavior (TPB) has been shown to have greater predictive and explanatory capacity compared to the Theory of Rational Behavior and offers a more flexible approach to incorporating additional variables that may have explanatory and predictive effects on specific behaviors and behavioral intentions [22]. Therefore, TPB has been chosen as the theoretical framework for investigating the self-disclosure behavior of Chinese COVID-19 patients on social media. To supplement TPB, we will also integrate the Privacy Calculus Theory (PCT) to explore the factors that motivate self-disclosure on social media platforms.

### 2.2. Theory of Planned Behavior

The Theory of Planned Behavior (TPB) is a widely used theoretical framework in social psychology to predict and explain individual intentions and behavior [23]. It was first proposed by Fishbein in 1963 as the Fishbein Model [24], which emphasized the role of attitudes in determining behavioral intentions. In 1975, Fishbein and Ajzen introduced the *Theory of Reasoned Action* [25], which added subjective norms as an influencing factor to the multi-attribute attitude theory, stating that attitudes and subjective norms jointly influence behavioral intentions. However, this theory assumes that individuals are rational and their behavior is entirely under their control. In 1985, Ajzen expanded on this model and introduced the concept of perceived behavioral control to create the TPB [26], which highlights the combined influence of attitudes, subjective norms, and perceived behavioral control on behavioral intentions. In 1991, he published the paper *Theory of Planned Behavior* [23], marking the maturity of the theory.

TPB not only takes into account individual internal factors but also incorporates external social factors, allowing this model to consider both the individual and the social environment where people live [27]. According to Ajzen, TPB can be adapted to include any variable that may explain or predict behavior and intentions. TPB has become one of the fundamental theories for studying individual behaviors due to its capacity to explain and predict specific behavioral intentions and behavioral decisions [28]. This flexibility has made TPB a popular framework in various fields, including sociology, psychology, management, economics, and communication, where it has been applied to study diverse behaviors such as consumer behaviors, privacy-protective behaviors [29], and self-disclosure behaviors [30].

In the field of health communication research, TPB is recognized as a highly effective framework for predicting and explaining health behaviors. It is considered one of the most popular, concise, and predictive causal models for health behavior [31]. TPB has been extensively applied in studies related to various health behaviors, including alcohol consumption [32], tobacco use [33], healthy eating [34], physical exercises [35], health screening [36], and health information sharing [37]. During the COVID-19 pandemic, TPB has been frequently employed to investigate behaviors related to pandemic control [38], vaccination [39], physical exercises [40], healthy eating [41], health protective measures [42], health information-sharing [43], and other behaviors.

**Figure 1:**
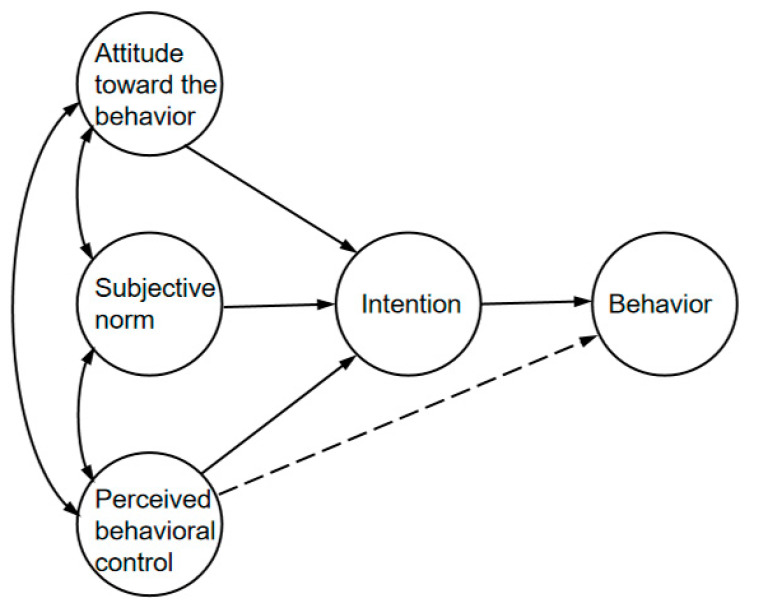
Theory of Planned Behavior model.

TPB consists of five elements: Attitude toward the Behavior (AB), Subjective Norm (SN), Perceived Behavioral Control (PBC), Behavioral Intention (BI), and the actual behavior (B). Individual behavioral intentions are jointly influenced by attitudes, subjective norms, and perceived behavioral control [44], whereas self-efficacy can directly impact actual behavior in specific situations, similar to the potential effect of intention, as illustrated in Figure 1.

#### 2.2.1. Behavioral Attitudes and Privacy Calculus Theory

In TPB, attitudes toward a behavior refer to individuals’ positive or negative evaluation of a specific behavior. The more positive the attitude, the greater the behavioral intention, and vice versa [23]. However, due to the private nature of health information, most studies on self-disclosure have incorporated the Privacy Calculus Theory (PCT) [14,45]. According to PCT, individuals evaluate the benefits and risks [15] and make decisions based on their perceived cost–benefit analysis [46]. This perspective has been applied to explain self-disclosure decisions of social media users in various studies [47]. Many studies investigating self-disclosure in various contexts have combined TPB with PCT. This study similarly adopts a combined approach of the two theories to examine self-disclosure behavior among Chinese COVID-19 patients. The behavioral attitudes in this study were measured with the use of two variables, perceived benefits, and perceived risks, based on the principles of PCT. This approach allows for the customization of TPB different contexts and research questions by adding or removing variables.

##### Perceived Benefits

Research has revealed that the reasons behind social media users’ self-disclosure behaviors often relate to perceived benefits, such as the need to maintain [15] and develop relationships, seek entertainment [48], and reciprocate [49]. 

##### Perceived Risks

Studies on online health communities [50] have shown that patients may be hesitant to disclose personal information due to concerns about potential negative consequences such as stigma [51] in online communities [52,53]. Additionally, it has been found that users’ willingness to disclose personal information online can be negatively impacted by their perception of risks [54].

Based on the aforementioned research, the following hypotheses are proposed:

**H1:**  *The perceived benefits of disclosing medical history have a positive effect on the intention to disclose among COVID-19 patients.*

**H2:**  *The perceived risks of disclosing medical history have a negative impact on the intention to disclose among COVID-19 patients.*

#### 2.2.2. Subjective Norms

In TPB, subjective norms refer to the social pressures that individuals experience in relation to carrying out a particular action [25], whereby individuals are more likely to engage in a specific behavior if they perceive that significant others desire their engagement in such behavior [55]. Empirical evidence from numerous studies supports the influence of subjective norms on individual behaviors [56]. In the context of social media, users are exposed to the behaviors of others on the platform [55], and studies have shown that social norms [57,58] or peer pressure [15] can affect users’ self-disclosure behaviors.

Therefore, the following hypothesis is proposed:

**H3:**  *Subjective norms have a positive effect on the intentions to disclose medical history among COVID-19 patients.*

#### 2.2.3. Perceived Behavioral Control and Self-Efficacy

Perceived behavioral control is a multidimensional concept consisting of two components: the perceived ease or confidence of an individual to perform a specific behavior, and the degree to which the individual performs that behavior [59]. Kraft et al. (2019) found that individuals’ perceived behavioral control significantly affects their behavioral intentions [60]. In other words, the stronger the perceived behavioral control, the greater the behavioral intentions. Moreover, individuals with higher perceived behavioral control are more willing to disclose personal information. Ajzen (1991) proposed that perceived behavioral control can directly influence behavior in specific situations, which complements the potential influence of the intentions [22].

Bandura introduced the concept of self-efficacy (SE), which refers to an individual’s belief in their ability to perform specific behaviors [61]. The theoretical implications of self-efficacy are similar to those of perceived behavioral control [62]. Individuals’ self-efficacy has been found to influence their behavioral choices and willingness to take action [63]. Studies on health-related issues have confirmed its substantial influence on individuals’ behavioral intentions, such as willingness to get vaccination [64,65] and quit smoking [66]. In the context of self-disclosure, it has been revealed that self-efficacy in social media contexts has a positive effect on self-disclosure intentions and behaviors [67,68].

Based on the literature reviewed above, we propose the following hypotheses:

**H4:**  *Self-efficacy in disclosing medical history has a positive effect on the intentions to disclose medical history among COVID-19 patients.*

**H5:**  *Self-efficacy of disclosing medical history has a positive effect on the behavior of disclose medical history among COVID-19 patients.*

#### 2.2.4. Behavioral Intentions

Behavioral intentions are the degree to which individuals plan to perform a specific behavior. The greater the individuals’ intention to engage in the behavior, the more likely they are to do so in the absence of specific environmental factors that may hinder their behavioral plans [69]. Behavioral intentions are widely regarded as the most direct determinant of the occurrence of behavior and have been empirically validated in studies across various topics, such as smoking [70], alcohol consumption [71], breastfeeding [72], and physical activity [73].

Therefore, we propose the following hypothesis:

**H6:**  *The intentions of COVID-19 patients to disclose their medical history have a positive effect on their disclosure behaviors.*

**Figure 2:**
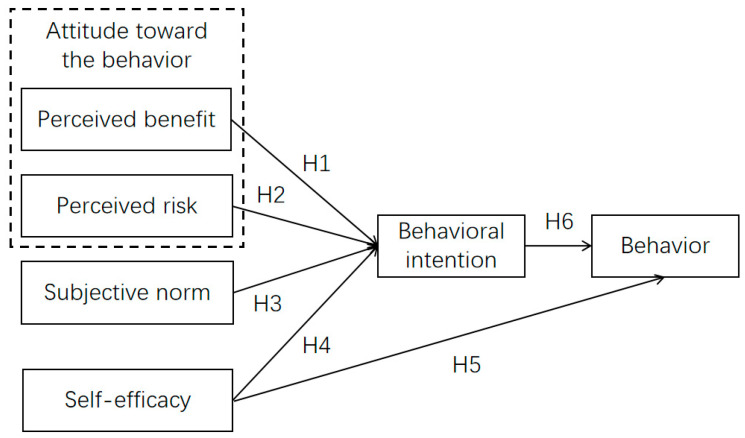
Research model.

Drawing on the previous analysis, we have integrated TPB with TPC to construct a theoretical model that explains the disclosure behaviors of Chinese COVID-19 patients pertaining their medical history. The subjective norms, self-efficacy, and behavioral intentions in the model were derived from TPB, while the perceived benefits and perceived risks were derived from TPC. The research model connecting all variables and hypotheses is illustrated in Figure 2, in which the six main variables and six hypotheses are included.

## 3. Method

### 3.1. Participants

To investigate the factors influencing self-disclosure on social media among Chinese COVID-19 patients, a questionnaire was administered to potential participants. The questionnaire included the questions “Have you ever been or are you currently infected with COVID-19? Have you posted this information on social media platforms?” Participants who responded that they were “not infected with COVID-19” were excluded from the study.

### 3.2. Data Collection

This study employed an online random questionnaire survey as the primary data collection method, which was administered through Questionnaire Star (www.wjx.cn, accessed on 30 March 2023), the largest online questionnaire platform in China. The survey was distributed via popular social media platforms such as WeChat, QQ, and Weibo. The data collection process was conducted in two stages, with strict measures in place to ensure the privacy of participants. 

In the first stage of data collection, a pre-survey was conducted, to help participants better understand the questions and fill out the questionnaire effectively. This also enabled the preliminary measurement of the questionnaire’s reliability and validity prior to the formal survey.

On 14 March 2023, the pilot survey was conducted to evaluate the reliability and validity of the questionnaire. A total of 111 questionnaires were collected via the Questionnaire Star platform, and 95 of them were deemed valid. Based on the results of the reliability and validity assessment, the questionnaire was then adjusted and modified.

The formal survey was carried out between 15 March and 21 March 2023. A total of 776 questionnaires were collected during this period, but some were considered invalid and excluded from the analysis due to short response times, repeated IP addresses, or failure to meet the participant requirements. After excluding these invalid samples, 593 valid questionnaires were obtained, yielding an effective response rate of 76.4%.

To meet the correlation coefficient requirements, the required sample size was estimated using G*Power 3.1.9.7 statistical analysis software, with one-way ANOVA and repeated measures ANOVA used. For the first calculation, one-way ANOVA was chosen with settings of 3 groups, an effect value of 0.25, statistical test power 1-β of 0.95, and a probability of one-class error α of 0.05, resulting in a minimum total sample size of 252. For the second calculation, the repeated measures ANOVA method was chosen with settings of the same group number, effect values, statistical test power, and the probability of one type of error as the first calculation, and a number of repeated measures of 2, resulting in a minimum total sample size of 189. Therefore, the minimum sample size required for this survey was concluded to be 252 [74]. This survey ultimately collected 593 valid questionnaire responses, which fulfilled the sample size requirement for the related analysis.

The demographic characteristics of the 593 valid participants are presented in Table 1. The study had a majority of participants, 66.1%, who were aged between 14–25 years, and 26.5% who were aged between 26–35 years. In terms of educational background, the majority of participants had obtained a bachelor’s degree or higher, with 52.9% having a bachelor’s degree and 35.9% having a master’s degree. In terms of occupation, the largest group of participants were students, accounting for 54.8%, followed by corporate employees at 22.1%. Regarding monthly income, 36.3% of the participants had a monthly income of ≤¥1500, and 18.4% had a monthly income of ¥1501–3000. Overall, the sample met the target group characteristics required for our study.

### 3.3. Measures

The questionnaire used in this study consisted of seven parts, namely participants’ demographic information, perceived benefits, perceived risks, subjective norms, self-efficacy, disclosure intentions, and behaviors. All independent variables were measured by a 5-point Likert scale (1 = strongly disagree, 2 = partially disagree, 3 = not sure, 4 = partially agree, 5 = strongly agree). In order to ensure the accuracy and validity of the questionnaire, a rigorous translation-back-translation procedure was implemented. 

All scales were derived from established scales in published academic papers. Given that the survey would be conducted in China and the participants are Chinese, a two-way translation method was employed to translate the English scales to suit the linguistic and cultural background of the participants. The scales were translated from English into Chinese (forward translation) and then translated back from Chinese into English (backward translation) by a Ph.D. researcher and an English-speaking master’s student, which could ensure accurate and unambiguous presentation of the questionnaires.

#### 3.3.1. Perceived Benefits

To evaluate perceived benefits, this study employed a scale consisting of five items adapted from Kehr F. et al. [75]. Sample items included “It is beneficial for me to disclose my COVID-19 status” and “It is convenient for me to disclose information that I have had or am having COVID-19”. The scale demonstrated good reliability with Cronbach coefficients of α = 0.858, M = 3.0398, SD = 0.89611.

#### 3.3.2. Perceived Risks

Voluntariness, knowledge, visibility, and trust [76] are among the characteristics of risk perception. As the perceived risks of disclosing medical history in public are related to the perceived risks of COVID-19 [77], this study used the COVID-19 Perceived Risk Scale (C19PRS) [78], a well-established scale, to measure the perceived risks of disclosing medical history among COVID-19 patients. The study selected five items based on the current social situation and pretest results in China, such as “I feel that I may get COVID-19” and “I am worried that I may get t COVID-19”. The scale demonstrated good reliability with Cronbach coefficients of α = 0.833, M = 3.9275, SD = 0.83265.

#### 3.3.3. Subjective Norms

To measure subjective norms of COVID-19 patients, this study used four items adapted from Ajzen and Fishbein’s research [25]. Sample items included “People who are important to me or have influence over me (e.g., family, leaders, teachers, colleagues, friends, etc.) think I should disclose information that I have had or am having the novel coronavirus”. The scale exhibited good reliability with Cronbach coefficients of α = 0.816, M = 3.3436, SD = 0.82577.

#### 3.3.4. Self-Efficacy

As self-efficacy is an individual psychological trait, this study used the general self-efficacy scale (GSES) to measure it based on previous studies [79]. The GSES was initially developed by Ralf Schwarzer et al. in 1981 and later refined into 10 items. Sample items include “I can cope with whatever happens to me” and “I can usually think of ways to cope when there is a trouble”. The scale demonstrated good reliability with Cronbach coefficients of α = 0.931, M = 3.5174, SD = 0.72101. 

#### 3.3.5. Behavioral Intentions

To measure the behavioral intentions to disclose medical history on social media, the privacy disclosure questionnaire developed by Cheng X. [80] and Choi H. [81] was used, consisting of four items. Sample items included “I am willing to disclose the information that I have been or am being infected with the novel coronavirus” and “I plan to disclose the information that I have been or am being infected with the novel coronavirus”. The scale demonstrated good reliability with Cronbach coefficients of α = 0.886, M = 3.4793, SD = 0.91976.

#### 3.3.6. Behaviors

For the measurement of the behaviors of disclosing medical history among COVID-19 patients, the dependent variable, i.e., the disclosure behaviors of medical history related to COVID-19, was recorded as a categorical variable. The measurement was “Have you ever been or are you currently infected with novel coronavirus? Have you posted this information on social media?” with three response options: 1 = No disclosure of disease information on social media; 2 = anonymous disclosure of disease information on social media; 3 = real name disclosure on social media.

### 3.4. Data Analysis Methods

The survey data was analyzed using the SPSS 26.0 and SPSS-AMOS 26.0 software packages. Descriptive statistical analysis, independent samples *t*-test, reliability analysis, one-way ANOVA, Pearson correlation analysis, and exploratory factor analysis were conducted using SPSS to thoroughly examine the data. Furthermore, validated factor analysis (CFA), structural equation modeling (SEM), and goodness-of-fit descriptions, including path coefficient (β), R^2^, f^2^, and Q^2^ were performed by AMOS to identify and determine potential relationships between variables.

## 4. Data Analysis Results

### 4.1. Differential Test of Demographic Characteristics

To examine the differences of demographic characteristics on the self-disclosure behaviors of COVID-19 patients on social media, we performed a one-way ANOVA with independent sample *t* -test using SPSS 26. Prior to the ANOVA, we conducted a chi-square test to ensure that the variance of each independent group was appropriate. A *p*-value less than 0.05 indicated that the variance was not chi-square, while a *p*-value greater than 0.05 indicated that the ANOVA test result was considered valid [82]. The results showed that only one control variable, namely gender, had a significant difference in the disclosure behavior of COVID-19 patients (*p*-value < 0.05), as illustrated in Table 2.

### 4.2. Correlation Analysis of Each Variable 

We conducted a Pearson correlation analysis on the six variables of interest in the present study, namely perceived benefits, perceived risks, subjective norms, self-efficacy, behavioral intentions, and behaviors. The purpose of the analysis was to explore the possible correlation among the variables and the strength of such correlation. The results revealed that perceived benefits were significantly and positively correlated with subjective norms, self-efficacy, behavioral intentions, and behaviors (*p* < 0.01); perceived risks were significantly and positively correlated with subjective norms, self-efficacy, and behavioral intentions (*p* < 0.01); subjective norms were significantly and positively correlated with self-efficacy, behavioral intentions, and behaviors (*p* < 0.01); self-efficacy was significantly and positively correlated with behavioral intentions (*p* < 0.01) and exhibited a positive correlation with behaviors (*p* < 0.05); behavioral intentions were significantly and positively correlated with behaviors (*p* < 0.01). These findings suggest that there exist potential interrelationships among the variables of the model, as displayed in Table 3.

### 4.3. Exploratory Factor Analysis and Confirmatory Factor Analysis

The references used in this study were selectively adapted to match the study’s scope, while also ensuring full attribution to the existing studies and established scales. These scales were validated through exploratory factor analysis (EFA) and confirmatory factor analysis (CFA). EFA was conducted on all scale items using SPSS 26.0. Firstly, the KMO value of the questionnaire content was measured to be 0.91 (>0.7), and Bartlett’s spherical test yielded *p* = 0.000 (<0.05), indicating a good fit for factor analysis. After conducting six rotations using principal component analysis and maximum variance rotation methods, we were able to identify five factors that had eigenvalues greater than 1, which accounted for a total variance of 66.12%, surpassing the desired threshold of 60%.

**Figure 3:**
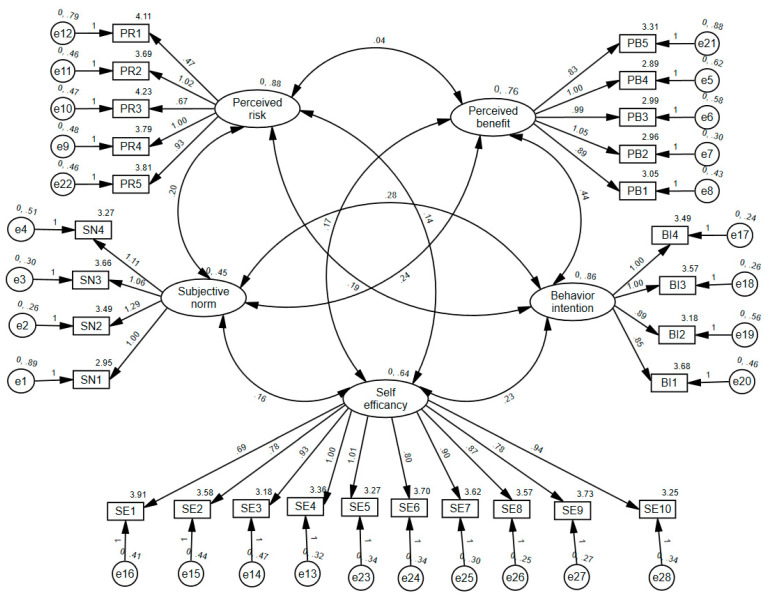
Confirmatory factor analysis. Ellipses indicate latent variables; boxes indicate observed variables, circles indicate measurement error.

We subsequently conducted confirmatory factor analysis (CFA) of the scale using AMOS 26.0. The results in Figure 2 show that the factor loadings of each item range from 0.56 to 0.851, all exceeding the cutoff of 0.5 (refer to Table 4). This indicates strong correlation and convergent validity for each latent variable measurement, suggesting that the questionnaire was constructed with reasonable and well-designed question items (refer to Figure 3).

### 4.4. Reliability and Validity Testing

The evaluation of item reliability in this study utilized Cronbach’s alpha as the standard. The Cronbach’s coefficients for each variable in Table 5 ranged from 0.816 to 0.931, all exceeding 0.8, which demonstrates the reliability and internal consistency of the measurement questions in this study. 

To test the validity of variables, this study measured content validity, convergent validity, and discriminant validity. To ensure content validity of the measurement items, established scales from previous research were used, and pre-surveys were conducted that yielded favorable results. 

The study’s f convergent validity was assessed through the factor loadings of each item, composite reliability (CR) of each latent variable, and the average variance extracted (AVE), which were all above the required thresholds as shown in Table 3 and Table 4. Specifically, all factor loadings of each item were above 0.5, CR of each latent variable was above 0.7, and AVE was above 0.5. These results indicate good convergent validity for all the items in this study. The discriminant validity of all items measured in this study was evaluated using the square root of the average variance extracted (AVE) value and the correlation coefficients between the other factors. As demonstrated in Table 6, the correlation coefficients between the latent variables were all less than the square root of AVE, indicating good discriminant validity.

The analysis above confirms the rationality and reliability of the questionnaire employed in this study. Further analyses involving structural model fitting (SEM) can be conducted to assess the rationality and validity of the model design.

### 4.5. Model Fitting

The structural equation model (SEM) was constructed using Amos 26, and its fit was evaluated based on the following recommended criteria for model fit, which included: (1) the relative chi-square (x2/df), with a suggested range of 1 to 5; (2) the root mean square error of approximation (RMSEA), with a suggested threshold of less than 0.08; (3) the standardized root mean square residual (SRMR), with a suggested threshold of less than 0.08; (4) the Tucker–Lewis index (TLI), with a suggested threshold of greater than 0.9; and (5) the comparative fit index (CFI), with a suggested threshold of greater than 0.9.

The initial evaluation results of the model showed some deficiencies. Specifically, the x2/df value was 3.653, the RMSEA value was 0.066, the SRMR value was 0.0621, the TLI value was 0.892, and the CFI value was 0.902. The TLI index did not meet the recommended threshold, suggesting that the model required revisions. 

To improve the model fit and address the TLI index deficiency, correction lines were added between the error variables based on the modification index (MI) of the initial model in Amos. The resulting model fit indexes after modification are presented in Table 7, which show that the x2/df value was 3.334, the RMSEA value was 0.063, the SRMR value was 0.0632, the TLI value was 0.902, and the CFI value was 0.912. All of these values met the recommended fit criteria, indicating that the model fit was significantly improved and was a good fit for the sample data.

### 4.6. Hypothesis Testing

**Figure 4:**
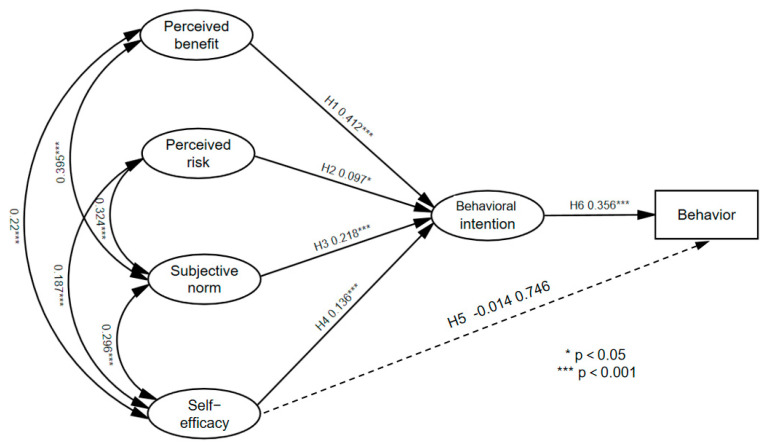
Path coefficients of the proposed model.

The structural equation model was employed in Amos 26 to examine the six paths in the study. Results were presented in the form of path coefficient plots and hypothesis testing outcomes, as shown in Figure 4 and Table 8, respectively. The results showed that the *p* values of H1, H2, H3, H4, and H6 were less than 0.05, indicating that these five paths had connectivity. Specifically, perceived benefits had a positive impact on disclosure intentions (β = 0.412, *p* < 0.001), thereby supporting H1. Perceived risks had a positive effect on disclosure intention (β = 0.097, *p* < 0.05), but H2 was not supported. Moreover, subjective norms had a positive influence on disclosure intentions (β = 0.218, *p* < 0.001), and H3 was supported. Likewise, self-efficacy positively influenced disclosure intentions (β = 0.136, *p* < 0.001), and H4 was supported. Disclosure intentions positively affected disclosure behaviors (β = 0.356, *p* < 0.001), thus H6 was supported. However, the effects of self-efficacy on disclosure intentions were not significant (β = −0.014, *p* = 0.746), and H5 was not supported. Overall, H1, H3, H4, and H6 were supported.

## 5. Discussion

### 5.1. Perceived Benefits, Perceived Risks Influence Behavioral Intention to Self-Disclose Medical History (H1, H2)

The results demonstrate that, in line with Hypothesis 1, the perceived benefits of disclosing medical history have a positive effect on the intentions to disclose it among COVID-19 patients. This finding is consistent with the study by Krasnvovah et al. [83], which explored self-disclosure and privacy calculus on social networking sites. According to the individual motivation theory, gain-like influences can be categorized into two types: extrinsic motivation, driven by external outcomes or goals, and intrinsic motivation, driven by internal satisfaction. Newly diagnosed patients disclosing their medical history on social media can receive both intrinsic and extrinsic benefits, including information-sharing, material support, and empathic care. Previous studies have shown that empathic care or social support can reduce the level of stigma and depression [84] among patients, thus yielding further benefits for them. As a result, the stronger the perceived benefits, the greater the intentions to disclose.

Contrary to the initial Hypothesis 2, it was found that the perceived risks of self-disclosure on social media had a positive influence on the intention to disclose among Chinese COVID-19 patients, which differs from the study by Tran T. et al. [85]. However, the “benefit-cost” theory suggests that in an emotionally connected social network, users are willing to take the risks of disclosing their medical history even though they are aware of the potential risks, and they are willing to pay a certain cost to obtain the desired benefits. Users’ perception of risks is immediate, without further considerations of past experiences or future risks, leading them to disclose their medical history for the sake of gain. Additionally, the “agenda melding” theory suggests that individuals align their personal agenda with that of a group to reduce cognitive dissonance and gain a sense of security and identity. The greater the perceived risk of COVID-19, the stronger the need to seek a sense of belonging, which leads to a greater intention to disclose medical history on social media.

According to Kurdry, any activity that revolves around significant media-related categories and boundaries can be classified as a media ritual [86], and self-presentation on social media is no exception. When COVID-19 patients publicly share their medical history on social media, they connect with a community of others who have gone through similar situations. Members of this community engage in shared discourse and emotional experience, amplifying the emotional energy of the interaction. This means that COVID-19 patients can participate in and interact with the ritual of disclosure whether they perceive benefits or risks in sharing their medical history on social media. Furthermore, the interaction can enhance the sense of social presence [87] and social existence of individuals using media, ultimately promoting their happiness [88,89].

### 5.2. Subjective Norms Have a Positive Influence on Behavioral Intentions to Self-Disclose Medical History (H3)

As demonstrated in Hypothesis 3, the subjective norms of disclosing medical history have a positive effect on the intentions to disclose it among COVID-19 patients. Chinese culture deeply values collectivism and emphasizes harmonious relationships between individuals. As a result, Chinese people tend to be more compliant with social norms [90]. Tajfel’s Social Identity Theory posits that individuals who belong to a collective are influenced by both the collective as a whole and its individual members. These individuals are motivated to adopt the emotional values and beliefs of the collective in order to establish a positive and consistent identity. Self-disclosure, especially on social media, plays a crucial role in fostering intimate relationships with others, including romantic partners, friends, and family [91]. The intention to perform a behavior such as self-disclosure is influenced by the subjective norms, which refer to individuals’ perceptions of the level of approval or support they receive from those around them or important to them. When a patient is diagnosed with a medical condition and his or her significant ones either disclose or suggest the disclosure of the patient’s medical history, the resulting subjective norm becomes a potent driving force behind the patient’s decision to disclose the medical information on social media. This can be attributed to the patient’s desire to establish a collective identity and/or fulfill their obligation to conform to the expectations and permission of their significant ones. The strength of the subjective norms correlates with the intensity of patients’ intentions to reveal their medical history on social media.

### 5.3. Self-Efficacy Has an Impact on Behavioral Intention to Self-Disclose Medical History (H4)

As shown in Hypothesis 4, self-efficacy is positively related to the intentions to disclose medical history among COVID-19 patients. Previous studies have revealed that individuals who contracted the virus during the early stages of the COVID-19 pandemic in China faced not only physical challenges but also psychological distress, social stigmatization, and discrimination associated with their illness [51]. This stigma was characterized by four dimensions: social exclusion, economic discrimination, intrinsic shame, and social isolation [92]. However, with effective public health policies implemented in China and the outbreak under control over time, the sense of disease stigma among COVID-19 patients gradually diminished. As the virulence of the virus decreased from late 2022 to early 2023, patients experienced less disruption to their daily lives and economic well-being. In addition, with an increase in the number of infected individuals, medical experts have shared the latest development and knowledge about the virus with the public, which reduced anxiety and improved understanding on this regard. This led to a more direct understanding of the virus for individuals who had contracted it or knew someone who had, thus resulting in a decreased sense of stigma across all dimensions and a greater sense of control over the infection. Individuals with higher levels of self-efficacy felt more confident and capable of handling potential issues that may arise from self-disclosure, making their intentions to disclose the medical history stronger.

### 5.4. Self-Efficacy Does Not Directly Influence the Behavior of Disclosing Medical History among COVID-19 Patients (H5)

Although Hypothesis 5 predicted a positive relationship between self-efficacy in disclosing medical history and the intentions to disclose among COVID-19 patients, the findings did not support this hypothesis. This could be attributed to the social media use patterns of neocon patients. Some users, despite having high self-efficacy in social media usage and self-disclosure, may not be accustomed to frequently disclosing all their personal situations on social media in their daily lives. Moreover, COVID-19 illness is still a sensitive topic, and the patients must consider various factors such as their physical conditions and the preferred self-presentation style on social media before deciding to disclose their medical history. Therefore, self-efficacy does not directly and significantly influence self-disclosure behavior in this population.

### 5.5. Self-Disclosure Intentions Have a Positive Impact on Disclosure Behaviors (H6)

As stated in Hypothesis 6, COVID-19 patients’ intentions to disclose their medical history have a positive effect on their disclosure behaviors. This is consistent with previous research that has found [93] a positive relationship between intentions and behaviors. Theoretical frameworks, such as Technology Acceptance Model, Theory of Rational Behavior, and Use and Satisfaction Theory, have highlighted the importance of intentions as a crucial precursor to the occurrence of behaviors. However, there are instances where individuals express a strong intention to disclose their medical history but do not follow through with the behavior, leading to a discrepancy between intentions and behaviors. This paradox may be attributed to differences in personality traits among users. Individuals with an open or extroverted personality may consider disclosure a prerequisite for social engagement and, therefore, may disclose more frequently [94,95]. In contrast, those with an agreeable personality may prioritize trust and altruism over self-disclosure intentions, leading them to limit the information they want to share to avoid conflicts. These differences in personality traits can result in varied levels of disclosure among individuals [96].

## 6. Conclusions

Drawing upon the evolving landscape of the COVID-19 pandemic in China and its associated cultural norms, a trend has emerged where COVID-19 patients have transitioned from concealing their medical history to voluntarily disclosing it on social media. The present study aims to examine the influence of perceived risks, perceived benefits, subjective norms, and self-efficacy on patients’ behavioral intentions to disclose their COVID-19 medical history, as well as the influence of the intentions on their actual disclosure behaviors on social media. By combining the Theory of Planned Behavior and the Privacy Calculus Theory, a structural equation model was constructed and analyzed. The results indicate that patients’ perceived risks, perceived benefits, subjective norms, and self-efficacy have positive effects on their intentions to disclose medical history of COVID-19. A positive relationship was found between the disclosure intentions and the disclosure behaviors, whereas self-efficacy was not found to have a direct impact on disclosure behaviors. 

In terms of theoretical significance, this study contributes to the application of TPB by incorporating PCT, introducing new constructs that improve the explanatory and predictive capacity of behavioral intentions, particularly in relation to the direct impact of perceived behavioral control on behaviors in specific contexts. 

In practical terms, scientific dissemination of health information is found to be more beneficial for individuals under Chinese cultural values of collectivism to enhance their understanding of disease and related health behaviors, thereby helping them overcome panic and disease stigma.

To respond to the evolving epidemic situation, public health departments of government should adjust prevention and control policies and provide timely, accessible, and scientific popularization and publicity. This can be achieved through easy-to-understand methods, disseminating information on disease prevention and treatment to different groups to enhance public understanding and familiarity, thereby reducing disease stigma, uncertainty, and psychological pressure, alleviating public perceived risks, and enhancing public self-efficacy. Experts or opinion leaders can also be invited to share their knowledge and experiences about the disease on social media and other platforms to create a supportive environment, thus making the public more positive toward the disease and patients more encouraged to disclose their illness. In addition, specialized platforms or social media communities can be established to encourage disclosure and sharing, enhance patients’ perceived benefits, and facilitate active public communication to convey accurate and comprehensive information, and promote health behavior practices, with ensuring user privacy as a prerequisite.

## 7. Limitations and Prospects

There still exist some limitations in this study that require further explorations in future research. Firstly, the sample was limited to youth as they tend to rely more heavily on social media and are more willing to disclose information on these platforms, which may have biased the results of this study. Future research could investigate the disclosure behaviors of social media users with lower levels of media exposure, the middle-aged and elderly individuals, and the factors that influence disclosure behaviors such as social media usage habits and exposure levels. This would specify the research objects and broaden the scope of research, and also provide additional perspectives on social media self-disclosure. Secondly, this study only considered two types of disclosure behavior: real-name disclosure and anonymous disclosure. Future research could investigate different types of disclosure behaviors, as well as disclosure of different types of information and social media platforms to explore the similarities and differences in users’ intentions to disclose personal information of varying degrees, the impact of the nature and functions of different platforms on users’ disclosure intentions and behaviors, or to study cross-platform disclosure behaviors and the deployment of different platforms. Thirdly, a deviation rate was observed between participants’ behavioral intentions and their actual behaviors. Further studies could explore the reasons for this deviation such as the effect of each factor on the paradox or the underlying mechanism of induction through binary logit, NCA, fsQCA, or in-depth interviews. Finally, other variables, such as personality traits, level of desire [97], safety attitude [98], and flows [99], may also influence self-disclosure intention and behaviors. Future studies may incorporate these variables to refine the model and improve its explanatory capacity.

## Figures and Tables

**Table 1:** Demographic characteristics of participants (*n* = 593).

Characteristics	Demographic Information	Frequency	%
Gender	Male	212	35.8
	Female	381	64.2
Age	14 and below	1	0.2
	15–24	392	66.1
	25–34	157	26.5
	35–44	17	2.9
	45–54	23	3.9
	55 and above	3	0.5
Education	Primary school and below	4	0.7
	Junior High School	10	1.7
	Senior High School	20	3.4
	Junior college	30	5.1
	Bachelor’s degree	313	52.8
	Master’s degree	213	35.9
	Doctoral degree and above	3	0.5
Profession	Student	325	54.8
	Professional skill worker	31	5.2
	Civil servant or public institution personnel	39	6.6
	Corporate employees	131	22.1
	Self-employed	11	1.9
	Freelance	17	2.9
	Not employed/unemployed	25	4.2
	other	14	2.4
Monthly Income (CNY)	≤1500	215	36.3
	1501–3000	109	18.4
	3001–4500	68	11.5
	4501–6000	67	11.3
	6001–7500	46	7.8
	≥7501	88	14.8

“CNY” refers to the China Yuan.

**Table 2:** Differential testing of demographic characteristics in behavior (*n* = 593).

Characteristics	Demographic Information	Mean	SD	t, F, or r	*p*-Value
Gender *	Male	1.62	0.897	8.634	0.003
	Female	1.8	0.941		
Age	14 and below	1	0	1.075	0.373
	15–24	1.77	0.94		
	25–34	1.73	0.917		
	35–44	1.65	0.931		
	45–54	1.35	0.775		
	55 and above	1.67	1.155		
Education	Primary school and below	1	0	1.164	0.324
	Junior High School	1.4	0.843		
	Senior High School	1.45	0.826		
	Junior college	1.63	0.89		
	Bachelor’s degree	1.75	0.935		
	Master’s degree	1.78	0.942		
	Doctoral degree and above	2	1		
Profession	Student	1.77	0.945	0.34	0.935
	Professional skill worker	1.71	0.902		
	Civil servant or public institution personnel	1.79	0.951		
	Corporate employees	1.68	0.905		
	Self-employed	1.82	0.982		
	Freelance	1.65	0.931		
	Not employed/unemployed	1.68	0.945		
	other	1.5	0.855		
Monthly Income (CNY)	≤1500	1.68	0.923	0.927	0.463
	1501–3000	1.8	0.96		
	3001–4500	1.6	0.866		
	4501–6000	1.72	0.918		
	6001–7500	1.85	0.965		
	≥7501	1.85	0.941		

Variables with “*“ indicate the heterogeneity of variance, using the Welch method. SD is the standard deviation.

**Table 3:** Correlation analysis among variables.

	PB	PR	SN	SE	BI	M	SD
Perceived Benefit	1					3.040	0.896
Perceived Risk	0.061	1				3.928	0.833
Subjective Norm	0.397 **	0.261 **	1			3.344	0.826
Self-efficacy	0.240 **	0.202 **	0.294 **	1		3.517	0.721
Behavioral Intention	0.510 **	0.209 **	0.445 **	0.305 **	1	3.479	0.920

** *p* < 0.01.

**Table 4:** Factor loadings.

Variables	Items	Factor Loadings
Perceived benefit	PB1	0.749
	PB2	0.851
	PB3	0.789
	PB4	0.787
	PB5	0.649
Perceived risk	PR1	0.56
	PR2	0.838
	PR3	0.773
	PR4	0.814
	PR5	0.796
Subjective norm	SN1	0.577
	SN2	0.801
	SN3	0.772
	SN4	0.821
Self- efficacy	SE1	0.647
	SE2	0.698
	SE3	0.745
	SE4	0.823
	SE5	0.816
	SE6	0.771
	SE7	0.814
	SE8	0.817
	SE9	0.787
	SE10	0.802
Behavioral intention	BI1	0.8
	BI2	0.664
	BI3	0.819
	BI4	0.811

**Table 5:** Reliability and convergence validity testing results.

Latent Variables	Cronbach’s Alpha	AVE	CR
Perceived benefit	0.858	0.561	0.864
Perceived risk	0.833	0.509	0.832
Subjective norm	0.816	0.557	0.832
Self-efficacy	0.931	0.579	0.932
Behavioral intention	0.886	0.670	0.890

CR is the composite reliability; AVE is the average variance extracted.

**Table 6:** Discriminant validity testing results.

	Perceived Benefit	Perceived Risk	Subjective Norm	Self-Efficacy	Behavioral Intention
Perceived Benefit	**0.749**				
Perceived Risk	0.039	**0.714**			
Subjective Norm	0.235 ***	0.206 ***	**0.746**		
Self-efficacy	0.165 ***	0.137 ***	0.16 ***	**0.761**	
Behavioral Intention	0.438 ***	0.179 ***	0.282 ***	0.229 ***	**0.818**

The bold values indicate the square root of AVE. *** *p* < 0.001.

**Table 7:** Model fitting indexes after modification.

Indexes	x2/df	RMSEA	CFI	TLI	SRMR
Observed value	3.405	0.064	0.915	0.905	0.0631
Ideal value	<5	<0.08	>0.9	>0.9	<0.08

RMSEA, the root means square error of approximation; CFI, comparative fit index; TLI, Tucker–Lewis index; SRMR, standardized root means square residual.

**Table 8:** Hypothesis testing results.

Hypothesis	Model Paths	Path Coefficients (β)	*p*-Values	Results
H1	Perceived Benefit → Behavioral Intention	0.412	***	Supported
H2	Perceived Risk → Behavioral Intention	0.097	0.021	Not supported
H3	Subjective Norm → Behavioral Intention	0.218	***	Supported
H4	Self-efficacy → Behavioral Intention	0.136	***	Supported
H5	Self-efficacy → Behavior	−0.014	0.746	Not supported
H6	Behavioral Intention → Behavior	0.356	***	Supported

*** *p* < 0.001.

## Data Availability

The raw data supporting the conclusions of this article will be made available by the authors, without undue reservation.
